# MACC1和c-met在非小细胞肺癌中的表达及其预后价值

**DOI:** 10.3779/j.issn.1009-3419.2012.07.02

**Published:** 2012-07-20

**Authors:** 兴胜 胡, 曦 付, 世民 文, 心怡 邹, 雨松 刘

**Affiliations:** 1 637900 南充，川北医学院第二临床学院肿瘤科 Department of Oncology, the Second Affiliated Hospital of North Sichuan Medical College, Nanchong 637900, China; 2 618400 什邡，什邡市妇幼保健院检验科 Department of Clinical Laboratory, Maternal and Child Care Service Centre of Shifang, Shifang 618400, China; 3 618400 什邡，什邡市人民医院检验科 Department of Clinical Laboratory, The People' s Hospital of Shifang, Shifang 618400, China

**Keywords:** 肺肿瘤, MACC1, c-met, 浸润, 转移, 预后, Lung neoplasms, MACC1, c-met, Invasion, Metastasis, Prognosis

## Abstract

**背景与目的:**

已有的研究表明结肠癌转移相关基因1（metastasis-associated in colon cancer 1, MACC1）是一个与肿瘤浸润转移相关的新基因，该基因能够调节肝细胞生长因子受体（hepatocyte growth factor receptor, c-met）的表达。本研究旨在探讨MACC1和c-met在非小细胞肺癌（non-small cell lung cancer, NSCLC）组织中的表达及其与浸润转移和预后的关系。

**方法:**

采用免疫组化检测103例NSCLC组织及40例癌旁正常组织中MACC1和c-met蛋白的表达。

**结果:**

MACC1和c-met在NSCLC组中的阳性表达率均明显高于正常肺组织（*P* < 0.001）。MACC1和c-met阳性率均与肺癌的分化程度、T分期、淋巴结转移和TNM分期相关（*P* < 0.05），而与性别、年龄、吸烟及组织学类型等无关（*P* > 0.05）。MACC1和c-met的表达呈正相关（*r*=0.403, *P* < 0.001）。*Kaplan-Meier*生存曲线显示MACC1和c-met阳性组5年生存率均明显低于阴性组（*P* < 0.05）。*Cox*多因素分析显示MACC1是NSCLC的独立预后因素（*P*=0.026）。

**结论:**

MACC1和c-met的表达与肺癌的分化、浸润和转移密切相关，两者均对生存期有一定的影响，MACC1是NSCLC的独立预后危险因素。

## Abstract (en)

**Background and objective:**

It has been proven that metastasis-associated in colon cancer 1 (MACC1) is a new gene that is related to the invasion and metastasis of tumors. MACC1 also regulates c-met expression. The aim of this study is to explore the expressions of MACC1 and hepatocyte growth factor receptor (c-met), and its relationship with invasion, metastasis, and prognosis of non-small cell lung cancer (NSCLC).

**Methods:**

MACC1 and c-met expressions were detected in 103 cases of NSCLC and 40 cases of neighboring normal lung cancer tissue using immunohistochemistry.

**Results:**

MACC1 and c-met expressions were significantly higher in lung cancer tissues than that in neighboring normal tissue (*P* < 0.001). MACC1 and c-met expressions were associated with poor differentiation, advanced T stages, lymph node metastasis, and advanced TNM stages (*P* < 0.05) of NSCLC, but not with sex, age, smoking, and histological classification (*P* > 0.05). In addition, a positive correlation between MACC1 and c-met expressions was observed (*r*=0.403, *P* < 0.001). The result from the *Kaplan-Meier* survival analysis showed that the five-year survival rate in patients with positive MACC1 and c-met expressions was remarkanly lower than that in patients with negative expressions (*P* < 0.05). The result from the *Cox* regression analysis showed that MACC1 expression was an independent prognostic factor for NSCLC (*P*=0.026).

**Conclusions:**

MACC1 and c-met have an important function in the differentiation, invasion, and metastasis of NSCLC. MACC1 and c-met have poor prognosis in patients with NSCLC. Moreover, MACC1 expression is an independent prognostic factor for NSCLC.

目前，肺癌死亡率已位居男、女性恶性肿瘤的第一位^[1]^，全球每年至少有160万的新发病例和130万的死亡病例。非小细胞肺癌（non-small cell lung cancer, NSCLC）约占肺癌总数的80%。侵润和转移是恶性肿瘤最重要的生物学特征之一，亦是导致患者死亡的主要原因。肺癌易发生浸润和转移，其5年生存率低。目前有关肺癌浸润转移的机制尚不完全清楚，因此探索与肺癌浸润转移尤其是与预后相关的生物学指标，显得尤为重要。研究^[2]^表明，结肠癌转移相关基因1（metastasis-associated in colon cancer 1, MACC1）是2009年新发现的，能够预测肿瘤浸润转移及预后的新基因，其机制可能与调节肝细胞生长因子（hepatocyte growth factor，HGF，又称离散因子）及其受体c-met表达有关。为此，我们采用免疫组化联合检测了MACC1和c-met在NSCLC组织中的表达情况，并分析了它们与肺癌临床病理特征及预后的关系。

## 1 材料与方法

### 1.1 材料

选取2003年1月-2006年1月在川北医学院第二临床学院胸心外科手术标本103例和同期40例距离癌肿边缘5 cm以上的正常组织。所有患者术前均未化疗或放疗，有完整的临床资料和明确的术后病理诊断。其中男性77例，女性26例； < 60岁51例，≥60岁52例；不吸烟42例，吸烟61例；鳞癌56例，腺癌47例；高分化15例，中分化68例，低分化20例；T1期+T2期78例，T3期+T4期25例；N0期70例，N1, 2, 3期33例；Ⅰ期56例，Ⅱ期12例，Ⅲ期35例。

随访：所有患者每3个月均进行电话、门诊或住院部随访，随访开始于2003年4月，2011年1月结束，最短随访时间为4个月，最长随访时间为5年。期间有9例失访。

### 1.2 免疫组化检测MACC1和c-met蛋白的表达

手术标本经10%甲醛固定后，常规石蜡包埋、切片，厚度4 μm。采用免疫组化SP法（兔抗人MACC1多克隆抗体购自美国Sigma公司，兔抗人c-met多克隆抗体购自北京博奥森生物技术有限公司，免疫组化二抗SP试剂盒购自北京中杉生物公司）。用PBS液代替一抗作为阴性对照，按照试剂说明书进行操作。结果判断：所有切片均采用双盲法由两位病理科医师独立阅片。MACC1和c-met阳性表达均定位于细胞浆和细胞膜，呈浅黄色、黄色或棕黄色。随机选择10个高倍镜视野（400倍），每个视野连续计数100个细胞，共计数1, 000个细胞。最后表达以染色强度和阳性细胞率的得分之和进行判断：无染色记0分，弱染色记1分，中等染色记2分，强染色记3分；阳性细胞率 < 5%记0分，5%-25%记1分，26%-50%记2分， > 50%记3分。上述两项评分相加， < 3分为阴性，≥3分为阳性。

### 1.3 统计学处理

采用SPSS 17.0统计学软件包分析。率的比较采用χ^2^检验或*Fisher*精确检验，单因素生存分析采用*Kaplan-Meier*生存曲线和*Log-rank*检验，多因素生存分析采用*Cox*多因素分析模型，以*P* < 0.05为差异有统计学意义。

## 2 结果

### 2.1 MACC1和c-met蛋白检测结果

MACC1和c-met在癌组中的阳性率分别为67.9%（70/103）和68.9%（71/103），均明显高于其在正常组织中的表达5.0%（2/40）（χ^2^=45.684, *P* < 0.001）和7.5%（3/40）（χ^2^=43.545, *P* < 0.001）（图 1）。

**1:**
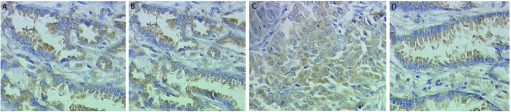
免疫组化检测MACC1在肺鳞癌（A）和腺癌（B）以及c-met在肺鳞癌（C）和腺癌（D）组织中的阳性表达（×400） The positive expression of MACC1 in lung squamous carcinoma (A) and adenocarcinoma (B), c-met in lung squamous carcinoma (C) and adenocarcinoma (D) tissue by immunohistochemistry (×400).

### 2.2 MACC1蛋白表达与肺癌临床病理特征的联系

统计学结果显示，MACC1和c-met的阳性率均随肿瘤分化程度的降低、T分期的增加、淋巴结转移和TNM分期的增加而增加（*P* < 0.05），而与性别、年龄、吸烟及组织学类型等无关（*P* > 0.05）（表 1）。

**1:** MACC1和c-met的表达与NSCLC的临床病理特征联系 Correlation of MACC1 and c-met expression with clinicopathologic characteristics of NSCLC

Variable	*n*	MACC1+ (%)	*χ*^2^	*P*	c-met+ (%)	*χ*^2^	*P*
Sex			0.666	0.414		0.666	0.414
Male	77	56 (72.7%)			56 (72.7%)		
Female	26	21 (80.8%)			21 (80.8%)		
Age (year)			0.320	0.571		0.025	0.875
< 60	51	36 (70.6%)			37 (72.5%)		
≥60	52	34 (65.4%)			37 (71.2%)		
Smoking			1.195	0.274		0.006	0.938
No	42	26 (61.9%)			30 (71.4%)		
Yes	61	44 (72.1%)			44 (72.1%)		
Pathological type			0.064	0.800		0.604	0.437
Squamous carcinoma	56	30 (53.6%)			42 (75.0%)		
Adenocarcinoma	47	24 (51.1%)			32 (68.1%)		
Differentiation			5.536	0.019		4.044	0.044
Well and moderate	83	52 (62.7%)			56 (67.5%)		
Poor	20	18 (90.0%)			18 (90.0%)		
T stage			3.900	0.048		4.260	0.039
T1-T2	78	49 (62.8%)			52 (66.7%)		
T3-T4	25	21 (84.0%)			22 (88.0%)		
Lymphatic metastasis			4.282	0.039		4.059	0.044
N0	70	43 (61.4%)			46 (65.7%)		
N1-N2	33	27 (81.8%)			28 (84.8%)		
TNM stages			7.674	0.006		5.042	0.025
Ⅰ+Ⅱ	68	40 (58.8%)			44 (64.7%)		
Ⅲ	35	30 (85.7%)			30 (85.7%)		

### 2.3 MACC1和c-met相关性分析

经*Spearman*秩相关分析发现，在103例肺癌组织中MACC1和c-met的表达呈正相关，（*r*=0.403, *P* < 0.001）。

### 2.4 生存分析

*Kaplan-Meier*生存曲线显示，MACC1阳性组的5年生存率为19.5%，明显低于阴性组的59.9%（χ^2^=15.093, *P* < 0.001）。c-met阳性组5年生存率为25.4%，明显低于阴性组的48.3%（χ^2^=8.230, *P*=0.004）（图 2）。

**2:**
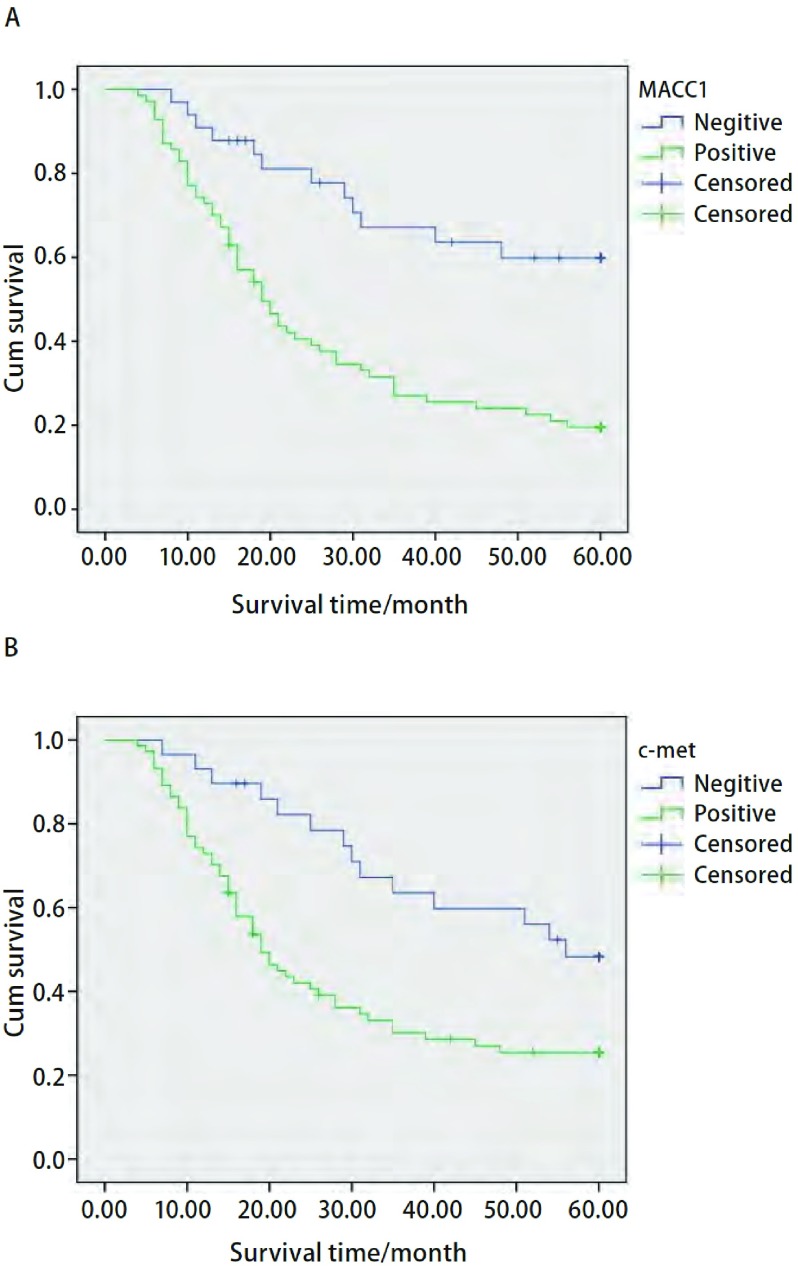
*Kaplan-Meier*累计生存时间曲线分析。A：MACC1表达阳性和阴性NSCLC患者；B：c-met表达阳性和阴性NSCLC患者。 *Kaplan-Meier* cumulative survival time curves analysis. A: MACC1 positive and negative expression group of NSCLC petients; B: c-met positive and negative expression group of NSCLC petients; NSCLC: non-small cell lung cancer.

### 2.5 *Cox*多因素回归分析

*Kaplan-Meier*单因素分析发现，分化程度、T分期、淋巴结转移、TNM分期均与患者的生存期相关，故均纳入*Cox*多因素分析模型。*Cox*多因素分析结果显示，只有MACC1（*P*=0.026）和TNM分期（*P*=0.004）是肺癌患者的独立预后危险因素，其相对危险度（relative risk, RR）分别为2.178（95%CI: 1.097-4.327）和2.230（95%CI: 1.288-3.860），而c-met表达不是患者的独立危险因素（*P* > 0.05）（表 2）。

**2:** *Cox*方程中的变量 *Cox* variables in the Equation

Variables	B	SE	Wald	df	Sig.	RR	RR (95%CI)	
							Lower limit	Upper limit
MACC1	0.779	0.350	4.943	1	0.026	2.178	1.097	4.327
c-met	0.236	0.338	0.487	1	0.485	1.266	0.653	2.455
TNM stages	0.802	0.280	8.209	1	0.004	2.230	1.288	3.860
Lymphatic metastasis	0.663	0.474	1.956	1	0.162	1.940	0.767	4.909
T stages	0.170	0.192	0.785	1	0.376	1.185	0.814	1.727
Differentiation	0.275	0.230	1.421	1	0.233	1.316	0.838	2.067
RR: relative risk.								

## 3 讨论

*MACC1*基因定位于人染色体7p21.1。该基因是2009年由Stein等^[2]^发现并命名的一个新基因，研究表明它与结肠癌的浸润、转移及预后密切相关。Stein检测到MACC1在结肠癌组织中表达异常增高，在有远处转移病例中其表达明显高于无转移者，MACC1高表达者5年生存率为15%明显低于低表达者的80%，且是结肠癌转移的独立预后指标。后来研究表明，MACC1在胃癌^[3]^、肝癌^[4-5]^、卵巢癌^[6]^、肺腺癌^[7]^、前列腺癌^[8]^等腺癌组织及膀胱移行细胞癌^[9]^、脑胶质瘤^[10]^、小细胞肺癌细胞系^[11]^等非腺癌组织中过表达，并且与浸润转移密切相关。MACC1高表达的肝癌组患者总生存率和无病生存率均明显低于低表达组^[5]^。在肺癌的研究中，Chundong等^[7]^采用免疫组化方法对197例手术后肺腺癌患者研究结果显示，MACC1的阳性率为65.5%，在复发组中的阳性率为82.5%（33/40），明显高于非复发组的61.1%（96/157），阳性组无病生存期较阴性组降低。杨淑慧等^[11]^观察到MACC1在小细胞肺癌细胞系中高表达，用MACC1 siRNA抑制其表达后，能明显抑制癌细胞的增殖迁移能力。

HGF是一种多功能细胞活性因子，具有很强的促有丝分裂作用，可诱导上皮细胞和成纤维细胞发生离散和运动。c-met是由原癌基因编码的HGF受体，主要在各种上皮细胞中表达。c-met编码了酪氨酸激酶，可调节肿瘤细胞侵袭性生长。HGF须与c-met受体结合方能发挥作用，两者结合导致两个酪氨酸残端在羧基端的磷酸化，引起一系列信号转导蛋白的酶促反应，从而调节相应的生物学行为，如细胞的运动等。正常的HGF/c-met通路在调节胚胎发育及组织损伤修复，而异常激活促进肿瘤的转移。HGF/c-met在多种恶性肿瘤组织中异常表达，并且与肿瘤的进展密切相关^[12, 13]^。

MACC1是HGF/c-met信号通路的一个关键调节因子。c-met已被证明是MACC1的转录子靶点^[2]^，它能通过扩增和（或）变异而不依赖与HGF的结合来激活。MACC1调节c-met的机制，可能是与c-met启动子区的SP1位点结合，从而激活c-met，促进其转录。HGF与c-met结合后，能启动下游3条信号途径：①磷脂酰肌醇3-激酶（PI3K）；②信号转导及转录激活因子（STAT）；③有丝分裂原活化激酶（MAPK）。这3条通路是细胞核内主要转录机构，从而促使肿瘤细胞增殖、运动、浸润、转移及血管生成等。另外，MACC1还可通过促进细胞外基质的降解、调节细胞骨架的结构^[8]^等方面，促进肿瘤的浸润和转移。

本研究结果显示，MACC1和c-met蛋白均在NSCLC组织中呈过表达。虽然文献多数报道MACC1在腺癌中高表达，但本研究首次报道了其在鳞癌组织中的高表达，与腺癌组织中的表达无统计学差异，这可能与MACC1（与c-met相关）在肿瘤的发展过程中普遍起作用相关，这已在膀胱癌^[9]^、脑胶质瘤^[10]^、小细胞肺癌细胞系^[11]^非腺癌中得到证实。本研究表明，MACC1和c-met蛋白的异常表达均与肺癌的分化程度、T分期、淋巴结转移和TNM分期密切相关，与文献报道基本一致。T分期是肿瘤体积的大小和侵袭能力的综合反应，提示MACC1参与了肿瘤的分化、增殖、侵袭和转移等。本研究中MACC1和c-met的表达呈正相关（*r*=0.403, *P* < 0.001），与Zhang^[6]^在卵巢癌中的报道（*r*=0.429, *P*=0.002）基本一致，但高于Qiu^[5]^在肝癌中对两者mRNA检测的报道（*r*=0.235, *P*=0.009），导致这种差异可能与肿瘤的类型、蛋白/mRNA检测的差异及样本量有关。本研究生存分析显示，MACC1和c-met的高表达均预示肺癌的不良预后，其中MACC1是肺癌独立预后危险因素，这与Stein^[2]^在结肠癌、Qiu^[5]^在肝癌及Chundong^[7]^在肺腺癌中的报道基本一致，提示MACC1可能是肿瘤的一个重要独立预后分子指标。

综上所述，我们的研究揭示了MACC1和c-met均在肺癌组织中过表达，其表达与肺癌的分化、浸润转移相关，它们对生存期均有一定的影响，而MACC1可能是肺癌的一项新的独立预后指标。因此，通过检测MACC1和c-met的表达可以更好的判断肺癌的恶性程度和预后，设想通过抑制MACC1的表达，抑制肿瘤浸润转移等，改善肺癌的不良预后，为肿瘤的靶向治疗提供新的靶点，为肿瘤发病机制的研究提供新的思路。
